# Gallium-based nascent electrode materials towards promising supercapacitor applications: a review

**DOI:** 10.1039/d3ra04537d

**Published:** 2023-08-15

**Authors:** Amtul Nashim, Ritik Mohanty, Priyadarshi K. Ray, K. M. Parida

**Affiliations:** a Centre for Nano Science and Nano Technology, Institute of Technical Education and Research, Siksha ‘O’ Anusandhan (Deemed to be University) Bhubaneswar 751019 India kulamaniparida@soa.ac.in

## Abstract

To meet the energy requirement of the modern era, supercapacitors are promising candidates for energy storage devices, which possess the potential to compete with the future battery technology. To accomplish this pivotal task, it is vital to choose electrode materials that have high power and energy density as well as superb electrochemical stability. For the past few years, the use of gallium-based materials for energy storage applications has attracted attention because of their excellent activity towards electrochemical energy storage applications despite the single oxidation state (*i.e.*, +3 which is redox inactive and does not contribute towards pseudo capacitance). Recently, research on gallium-based materials has started and will be continued further owing to the fact that gallium-based materials possess numerous excellent properties such as fast charge and discharge rate, high power density, long cycle life, stability over a wide range of temperatures, excellent electron velocity, superior chemical and physical stabilities and high voltage application capability, which make them a potential class of electrode materials for supercapacitors. The enhancement in the electrochemical performance upon the introduction of gallium into the system can make it a futuristic candidate for electrochemical energy storage devices. Herein, we systematically outline the synthesis and characterization of gallium-based materials and their composites as explored by esteemed researchers focusing only on their supercapacitive performance *via* electrochemical techniques. For a better understanding, the underlying charge storage mechanism and identified characteristics are presented to give a crystal-clear idea about the field. In addition, the key challenges and impending perspectives of gallium-based electrodes for supercapacitor applications are debated.

## Abstract 2

Gallium is present in abundance in earth crust and is considered as the environmentally friendly alternative to the many toxic and rare elements. Review summarizes the various categories of Ga based systems for supercapacitors.

## Graphical abstract

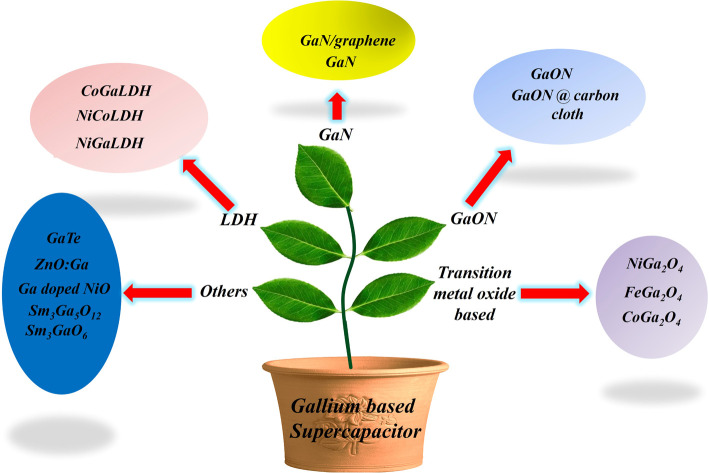

## 1. Introduction

**Fig. 1:**
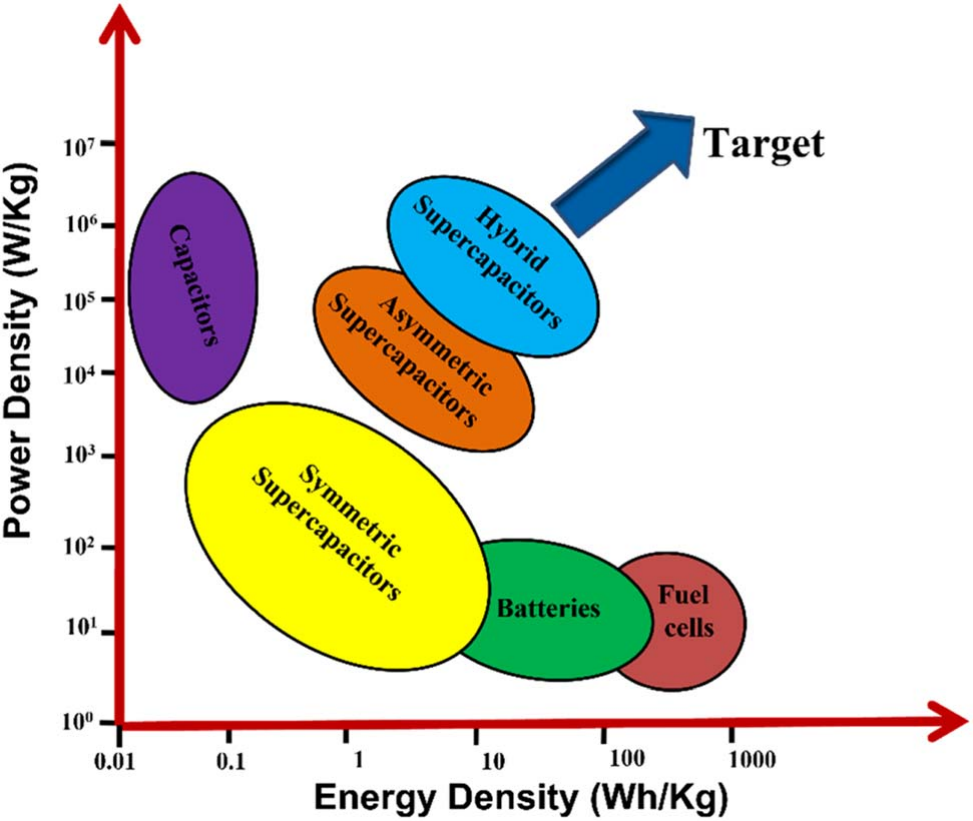
Ragone plot of different ESSs.

In the past few decades, many efforts have been made worldwide to shift our energy needs towards renewable sources such as tidal, hydro, solar and wind energies. Even though these resources are clean, sustainable and renewable, they are bound by natural or environmental conditions; hence, they show poor tenability and stability to generate electricity. Therefore, to fill this gap between energy generation and demand, reliable ESSs such as batteries, supercapacitors, and fuel cells are required.^1–3^Fig. 1 represents the Ragone curve explaining the relationship between the energy and power density of different ESSs. Among these, electrochemical capacitors, *i.e.*, supercapacitors, are considered future energy storage devices owing to their high energy density, high power density, cyclability, rapid charge discharge and capacitance performance. It is well known that supercapacitors store charge in two ways: (i) by electric double-layer capacitance (EDLC) and (ii) by surface redox reactions (pseudocapacitors).^1–3^ In 1853, Hermann Von Helmholtz, a physicist from German, discovered the idea of electrical double layer, with a charged electrode immersed in an electrolyte solution. After 100 years of this discovery, in 1957 H. I. Becker at General Electric company demonstrated and patented the charging–discharging phenomena and double-layer capacitance in a cell. In 1966, researchers at the Standard Oil Company (Ohio) developed the earliest commercial EDLC; they did not commercialize their technology and licensed to a Japanese multinational company, NEC (Nippon Electric Company, Limited), and they finally marketed the technology as supercapacitors for computer memory applications.^4–6^ Between 1975 and 1980, Brain Evans Conway performed extensive fundamental and advance work on RuO_2_ electrochemical capacitors and successfully defined the difference between the supercapacitor and the battery for electrochemical energy storage behavior. In 1982, Pinnacle Research Institute (PRI) developed the first supercapacitor having low internal resistance for military applications and marketed as “PRI ultracapacitor”. Some of the commercially available supercapacitors are examples of commercial supercapacitors from different manufacturers such as Yanasko, Panasonic, Maxwell, Asahi Glass, Vina Tech, JSR Miaro, Nesscap, Fuji, Loxus, Batscap, Skeleton, and APower.^3–6^ However, still researchers in numerous universities and companies are actively at work to improve the electrochemical properties of supercapacitors such as specific energy, specific power, number of cycles, and wide voltage window. The major commercial electrode material is carbon based, which is widely used directly or by modifying the electrode material. Activated carbons are the widely used electrode materials for EDLC supercapacitors due to their large surface area and porous morphology. However, due to rich micropores and poor conductivity, they did not meet the requirement of an ideal EDLC supercapacitor. In contrast, due to the high chemical stability, large surface area, good electrical conductivity, and large mechanical flexibility of graphenes, they can be considered as more promising electrode materials.^7,8^ After doping graphenes with surface functional groups such as O, N and S, the pseudocapacitive nature is induced, thereby improving the electrical conductivity and electroactive surface area to augment its storage performance.^9,10^ Usually transition metal oxides, transition metal hydroxides, transition metal sulphides and conducting polymers are used as electrode materials for pseudocapacitors. TMO, TMS and conducting polymers possess a higher theoretical capacitance than that of graphenes (525 F g^−1^). However, pseudocapacitive materials often suffer from low conductivity, degradation, limited reversibility, and poor rate capability. However, by introducing pseudocapacitive materials into a graphene matrix, the above-mentioned issues can be solved to some extent.^11–13^ Conducting polymers such as poly(3,4-ethylenedioxythiophene) (PEDOT), polyaniline (PANI), polypyrrole (PPy), and polyacetylene (PA) exhibit excellent conductivity and high theoretical capacitance, which are considered as promising pseudocapacitive materials for pseudocapacitors.^14–16^

**Scheme 1:**
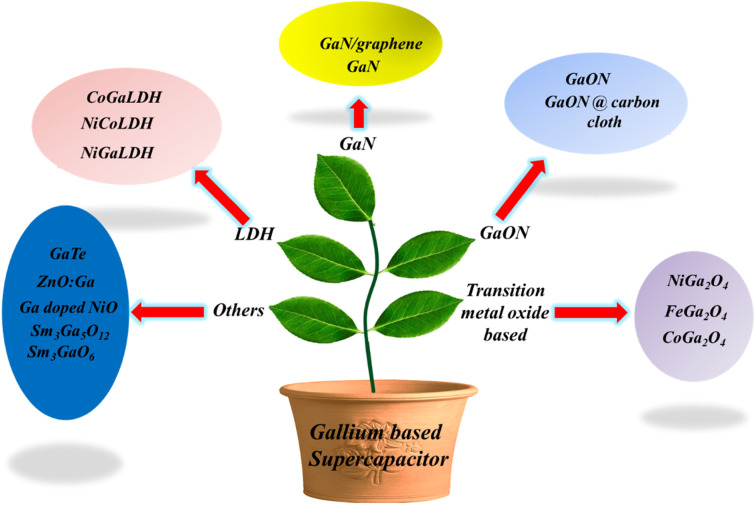
Various gallium-based systems for supercapacitors.

Post transition metal nanochalcogenides such as GaSe would be a great choice for photo/electrochemical and energy storage applications due to their suitable bandgap and low exciton binding energy.^17^ The oxidation state of Ga is 3+, and therefore, it does not take part in redox reactions, neither does it contribute anything towards pseudocapacitance. However, gallium-based materials show excellent activity towards electrochemical energy storage. Furthermore, Ga forms alloys with Li^+^ and gives maximum charge–discharge capacities compared to graphite or lithium titanate oxide.^18^ Therefore, Ga can be considered a good alternative to Si and Sn, as it possesses high current rate and the volume expansion is <60% of its original volume.^19^ FeGa_2_O_4_ is an example of battery-supercapacitor hybrid device, and this bimetallic oxide exhibits remarkable electrochemical storage performance due to the short ion diffusion path and good wettability of the electrolyte.^20^ In this review, the gallium-based electrode materials for supercapacitors are discussed owing to their unique properties. Gallium is a soft and silvery metal with a lower melting point, and it exhibits excellent electrochemical properties. Gallium-based materials possess high specific capacitance, *i.e.* they store a large amount of energy resulting in a higher energy density. Gallium-based materials show good conductivity and electrochemical stability, which means that they can withstand against the repeated charge discharge cycle ensuring the long cycle life of the supercapacitor. Moreover, gallium-based materials can be easily synthesized and modified to a desirable morphology. This tunability allows researchers to tailor the morphology, composition and structure of the electrode to optimize the performance of the semiconductor. With respect to the environmental aspects of the gallium-based electrode, gallium is present in abundance in the Earth's crust and considered an environmentally friendly alternative to many toxic and rare elements. By leveraging these advantages of gallium-based materials, this review summarizes various gallium-based materials for supercapacitor technology till date. In this review, gallium-based materials are categorized into 5 types, namely, GaN based, GaON based, LDH based, transition metal oxide based and others. Scheme 1 presents the various categories of gallium-based systems for supercapacitors.

## 2. Fundamental of supercapacitors

### 2.1 Basic charge storage mechanism of supercapacitors

**Fig. 2:**
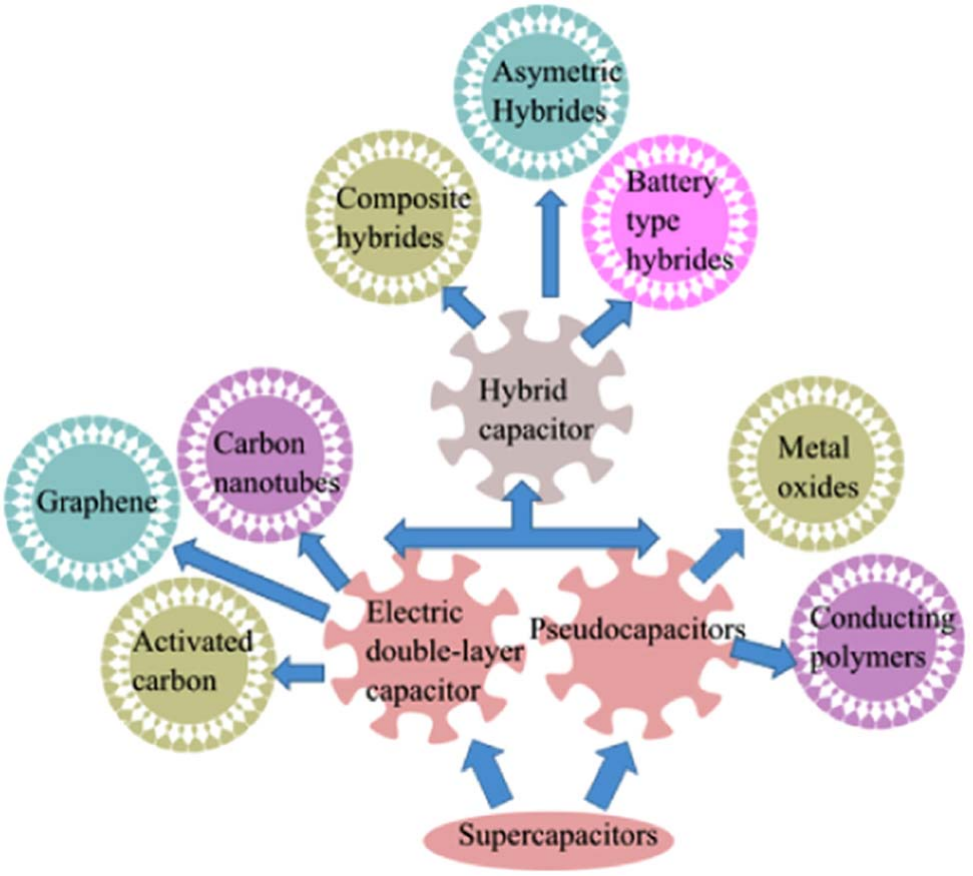
Types of supercapacitors (this figure has been reproduced from ref. 21 with permission from Elsevier, copyright 2018).

To address the ever-increasing energy storage issues, supercapacitors (SCs) can be used as an impressive solution. It is a quick current supplying system adopted to deliver high current and specific power density in less than one minute of duration. Subsequently, these devices can be operated alone or in combination with batteries to deliver augmented power and energy efficiency as well as prolonged cycling stability for future applications in various sectors. Generally, the ability of the SCs can be affected by factors such as electrochemical efficiency of the active materials, the choice of used electrolytes, and lastly, stable voltage window of the system. Thus, tremendous research attention has been paid to advance the field of SCs by fabricating innovative electrode materials with suitable structural and device designs to enable effective ion and electron transport with a short diffusion length. Furthermore, several types of SCs have already been used commercially, and they can be categorized by their definition of charging mechanisms or the materials used in the electrodes such as EDLC, pseudo, and hybrid, as shown in Fig. 2.^1–3,21^

Therefore, in this section, we briefly debate about the charge storage mechanism of the SCs, which mainly includes three types of capacitive behaviours, namely, EDLC, which uses the concept of pure electric charge accumulation at the interface of the electrode, PC, which arises from reversible and quick surface faradaic redox reactions, and hybrid nature, which takes advantage of both EDLC and PC mechanisms.

**Fig. 3:**
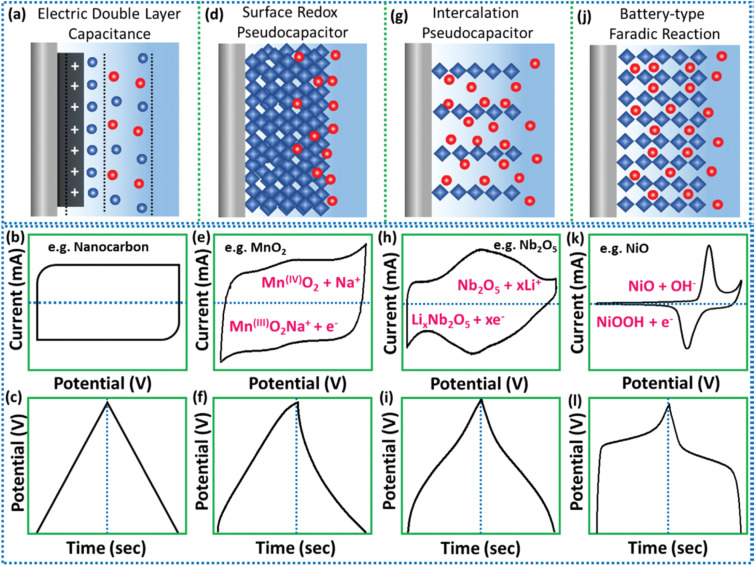
Schematic diagram of the diverse energy storage mechanisms with their equivalent signature CV and GCD curves for (a–c) EDLC, (d–f) surface redox, (g–i) intercalation type, and (j–l) faradic battery-type (this figure has been reproduced from ref. 22 with permission from Wiley, copyright 2002).

EDLC is the former and most studied SC fabricated using carbonaceous electrode materials such as activated carbons, graphenes, carbon nanofibers, carbon nanotubes and carbide-derived carbons. Here the capacitance arises (Fig. 3a) owing to the adsorption at the electrode/electrolyte interface due to the presence of both cations and anions in the electrolyte, enhanced specific surface area and improved conductivity. The aroused non-faradic capacitance sturdily depends on the surface-dominated properties of the carbon-based materials, which are directly accessible to the anions and cations. In the process of charging, the electrons travel towards the positive electrode from the negative electrode *via* the external connected loop, with anions traveling to the positive electrodes. Unlikely, the cations travel towards the negative electrode. In the discharging process, the traveling direction of electrons and ions is inverted. The signature CV and GCD curves of EDLC-based materials are displayed in Fig. 3b and c. Rectangular-box-type CV curves as well as symmetric triangular pattern GCD curves are assigned to the EDLC-type profile. The ultralow energy density values of the carbonaceous SC are the major hindrance for their effective commercialization. Thus, research focus is on the novel synthetic protocols to amplify the electrochemical performance of this class of materials.^1–3,22–24^

The term pseudocapacitance was first coined by Conway to define the electrode materials which display the CV and GCD similar to those of EDLC, but include different charge storing mechanisms. Unlike EDLC, the pseudocapacitive reactions occur at the electrode surface in faradic origin and store energy *via* quick and reversible redox reaction processes. Additionally, pseudocapacitive materials also display typical battery-type redox activities that occur at ultra-high rates like a capacitor, and reflect their electrochemical signature like quasi-rectangular CV curves along with quasi-triangular GCD curves. Overall, the charge storage mechanism includes two processes in PC materials, either surface redox reactions happening at the near surface of the material or intercalation-type reactions.^22–24^

In PC surface-redox reactions, the storage of electrical charge mainly relies on the redox reactions or charge transfers occurring at the active electrode surface. From the CV and the GCD curves depicted in Fig. 3d–f, we can conclude that generally surface-redox pseudo capacitors closely resemble (in Fig. 3a–c) those of carbonaceous materials, clearly demonstrating the linear dependency along with the whole potential window while storing the electrical charge. Therefore, from this we cannot conclude that pseudocapacitors store charges *via* the EDLC mechanism, but they can also store the charges *via* surface faradic and double-layer mechanism, which are called the intrinsic pseudocapacitive materials. Instead of surface redox reactions, few layered structured materials experience faradaic charge transfer owing to the ion intercalation into the layers without any crystallographic phase change. Especially, these particular kinds of materials show quick and reversible charge storage rates approaching or even superior to the traditional surface redox pseudocapacitive materials, thus they are not generally termed redox pseudocapacitors, instead they are called “intercalation pseudocapacitors”. This distinct term was coined by Dunn and Simon, which is a relative common trait in non-aqueous electrolyte systems.^22^ In this case, the electrochemical features are that current is directly proportional to the scan rate, but capacity does not change with charging time, and also the peak potentials do not considerably shift with change in sweep rate. Conversely to the EDLCs, here the process of charge storage is not restricted to the surface reaction and, thus, using bulk of the electroactive materials for the reversible faradic electrochemical reactions. The corresponding electrochemical profiles for distinguishability are displayed in Fig. 3g–i. Besides, the generally used battery-type materials are different from the capacitive-type materials in terms of the redox reaction mechanism, where the change of phase in active materials is responsible for the charge storage. Furthermore, *via* the process of charging and discharging, the voltage remains constant for the battery-type electrode, as shown in Fig. 3j–l. Thus, the active materials having solid-state diffusion-controlled faradic reactions generally called battery-type reactions show a pair of distinct redox peaks in CV curves along with a flat plateau like GCD curve similar to a battery displayed in Fig. 3k and l.^22^

To overcome the individual demerit offered by the EDLC and PC, hybrid supercapacitors are generally used, which use the polarizable electrodes made of carbon-based materials as well as non-polarizable electrode materials such as metals or conducting polymers. It involves both faradaic and non-faradaic reaction mechanisms to accomplish greater energy storage. Owing to the battery-type as well as capacitor-type electrodes, improved specific capacitance, excellent cycling stability, minimization cost and high efficiency are achieved.

The major categories of this material include asymmetric, composite and battery-type hybrid supercapacitors. Asymmetric hybrid supercapacitors are fabricated to fulfill high power and energy requirements simultaneously, as one electrode acts as capacitive and the other acts as faradic. In most of the cases, a carbon-based material generally works as a negative electrode, whereas a metal or metal oxide electrode operates as a positive electrode. The metal-based electrodes possess high volumetric capacity, which delivers increased energy densities, and the capacitive electrode provides decent cycling stability when equated with symmetric type SCs. However, in composite hybrid supercapacitors, the synergistic effects of high specific capacity, prolonged cycling stability, and high ion and electron conductivity are achieved. Here in the composite, the carbon offers a facile channel for charge transportation, whereas the metal oxide retains charge *via* a surface redox reaction. Lastly, in battery-type hybrids, one battery-type electrode and one capacitive electrode are used to provide effective charge storage performance.^1–3,22–24^

## 3. Application of gallium-based materials towards supercapacitors

In this section, the application of various gallium-based materials towards supercapacitors will be discussed. Table 1 represents the supercapacitive performance of various gallium-based materials.

**Table 1:** Represents the supercapacitive performance of various gallium-based materials

Catalyst	Synthesis method	Surface area (m^2^ g^−1^) and pore volume (m^3^ g^−1^)	Reaction conditions	Sp. capacitance (F g^−1^)	Cyclic stability (cycling number, cycling current density/scan rate)	Specific energy and specific power	Ref.
**GaN**							
GaN	Electrochemical etching process	22.2 m^2^ g^−1^	3 electrode system	23.67 mF cm^−2^ (at 0.01 V s^−1^)	99% (50 000, 10 mA cm^−2^)	45 mW cm^−2^	25
			C.E: Pt sheet				
		0.11 m^3^ g^−1^	R.E.: Hg/Hg_2_SO_4_				
			W.E.: stainless steel cloth				
GaN/GP	Chemical vapor deposition (CVD) method	24 m^2^ g^−1^	C.E: Pt sheet	237 mF cm^−2^ (at 0.1 mA cm^−2^)	98% (10 000, 5 mA cm^−2^)	0.30 mW h cm^−3^ (at 1000 mW cm^−3^)	26
			R.E.: Hg/Hg_2_SO_4_				
			W.E.: graphite paper				
			Electrolyte: 1 M H_2_SO_4_				
Porous GaN	Calcination	—	C.E: Pt sheet	21.22 mF cm^−2^ (at 0.1 mA cm^−2^)	99% (10 000, 5 mA cm^−2^)	0.58 μW h cm^−2^ (at 45 mW cm^−2^)	27
			R.E.: Hg/Hg_2_SO_4_				
			W.E.: stainless steel cloth				
			Electrolyte: 1 M H_2_SO_4_				
rGO-GaN nanocomposites	Chemical reduction	—	RE: Ag/AgCl	454 F g^−1^ (at 10 mV s^−1^)	75% (950, 5 A g^−1^)	—	29
			C.E.: platinum				
			Electrolyte: 1 M H_2_SO_4_				
GaN crystals	One-step ball milling process	—	W.E.: stainless steel cloth	52.58 mF cm^−2^ (at 0.8 mA cm^−2^)	86.2% (10 000, 8 mA cm^−2^)	13.3 mW h cm^−2^ (at 67.5 mW cm^−2^)	28
			Electrolyte: EMImNTf_2_				
Single-crystal GaN	Photoelectrochemical etching method	—	C.E: Pt foil	3.12 mF cm^−2^ (at 0.1 mA cm^−2^)	—	—	30
			R.E.: Hg/Hg_2_SO_4_				
			W.E.: stainless steel cloth				
			Electrolyte: 1 M H_2_SO_4_				

**LDH based**							
CoGa-LDH	Hydrothermal method	—	R.E.: HgO/Hg electrode	431.5 C g^−1^ (at 1 A g^−1^)	86.2% (8000, 15 A g^−1^)	68.07 W h kg^−1^ (at 825 W kg^−1^)	33
			C.E.: Pt plate				
			W.E.: Ni foam				
3D porous CoGa-LDH	Hydrothermal method	—	R.E.: SCE	0.62 C cm^−2^ (at 100 mV s^−1^)	97.5% (500, 31.5 mA cm^−2^)	33.38 W h kg^−1^ (at 10 088 W kg^−1^)	34
			C.E.: Pt sheet				
			W.E.: Ni foam				
NiCoGa-LDHs	Hydrothermal method	53.17 m^2^ g^−1^	C.E: Pt plate	2012.5 F g^−1^ (at 1 A g^−1^)	57.7% at 5.0 A g^−1^	84.22 W h kg^−1^ (at 800.1 W kg^−1^)	35
			R.E.: Hg/HgO				
			W.E.: carbon cloth				
			Electrolyte: 3 M KOH				
NiGa-LDH/N-GQD/NF	Hydrothermal method	229.26 m^2^ g^−1^	C.E: Pt plate	2160 F g^−1^ (at 1 A g^−1^)	87.5% (5000, 1 A g^−1^)	78.8 W h kg^−1^ (at 1432.7 W kg^−1^)	36
			R.E.: Hg/HgO				
			Electrolyte: 3 M KOH				

**GaON**							
GaON nanoparticles	Ammonolysis	45.3 m^2^ g^−1^	C.E: Pt foil	792 F g^−1^ (at 0.5 A g^−1^)	99% (10 000, 0.5 mA cm^−2^)		32
		0.012 m^3^ g^−1^	R.E.: Hg/Hg_2_SO_4_				
			Electrolyte: 1 M H_2_SO_4_				
GaON@carbon cloth	Moisture-assisted ammonolysis method	28.0 m^2^ g^−1^	C.E: Pt sheet	133 F g^−1^ (at 0.17 A g^−1^)	100% (10 000, 10 mA cm^−2^)	21.1 μW h cm^−2^ (at 0.5 mW cm^−2^)	31
		0.137 cm^3^ g^−1^	R.E.: Hg/Hg_2_SO_4_				
			Electrolyte: 1 M H_2_SO_4_				

**Transition metal oxide based**							
CoGa_2_O_4_/ZnFe_2_O_4_	Electrodeposition method followed by thermal annealing	—	C.E: Pt wire	232.2 F g^−1^ (at 50 A g^−1^) (device)	64% (6000, 50 A g^−1^)	82.56 W h kg−^1^	37
			R.E.: Ag/AgCl				
			Electrolyte: 2 M KOH				
CoGa_2_O_4_/graphene wrapped CuFeS_2_	Microwave-irradiation method, solvothermal, Hummer's route	104.3 m^2^ g^−1^	C.E: Pt	376.40 F g^−1^ (153.1 mA h g^−1^) (device)	93.7% (5000, 6 A g^−1^)	114.8 W h kg (at 750.4 W kg^−1^)	38
			R.E.: Ag/AgCl				
			W.E: nickel foam				
			Electrolyte: 6 M KOH				
CoGa_2_O_4_ 2D hexagonal nanoplates	Hydrothermal	65 m^2^ g^−1^	Electrolyte: 6 M KOH	1525 F g^−1^ (915 C g^−1^) (at 5 A g^−1^)	95 (10 000, 5 A g^−1^)	84 W h kg (at 1200 W kg^−1^)	39

**Other gallium based systems**							
GaTe	Vacuum induction melting method	18.012 m^2^ g^−1^	C.E: Pt wire	14 F g^−1^ (at 1 A g^−1^)	96% (10 000, 1 A g^−1^)	—	41
			R.E.: Ag/AgCl				
			W.E: graphite sheet				
			Electrolyte: 2 M KOH				
Ga incorporated NiO	Hydrothermal	2.64 m^2^ g^−1^	C.E: Pt foil	2.94 F cm^−2^	91% (10 00, 1 mA cm^−2^)	—	40
			R.E.: SCE				
			W.E: Ni foam				
			Electrolyte: KOH				
ZnO:Al	Hydrothermal treatment assisted by microwave	—	H_3_PO_4_/PVA electrolyte incorporated cellulose membrane sandwiched between electrodes. Copper paper as collector	284.6 F g^−1^	95.3%	56.9 W h kg^−1^	42
ZnO:Ga				118 F g^−1^	90.3%	23.9 W h kg^−1^	
ZnO:In				281.9 F g^−1^	91.6%	56.3 W h kg^−1^	
					After 500 cycles		
Sm_3_GaO_6_	Gel matrix method	—	C.E: Pt wire	103 mA h g^−1^ (1 A g^−1^)	82.65% (5000, 5 A g^−1^)	11.73 W h kg^−1^ (at 312.5 W kg^−1^)	43
			R.E.: Ag/AgCl				
Sm_3_Ga_5_O_12_			W.E: Ni foam	91.95 mA h g^−1^ (1 A g^−1^)	—	—	
			Electrolyte: 3 M KOH				

### 3.1 GaN-based supercapacitors

Owing to properties such as wide band gap (3.4 eV), high voltage capability and high melting point, GaN has been considered for many applications including optoelectronic, field emitter, sensing, and high-frequency devices. Additionally, GaN possesses numerous excellent properties such as high carrier concentration per volume, high thermal conductivity, excellent electron velocity, superior chemical and physical stability and high voltage capability, which make it a potential electrode material for supercapacitors. Recently, the wide band gap semiconductor has become an interesting research topic in the field of electrochemical energy storage devices. It is well known that GaN crystals have low surface area and poor conductivity, which reduce their efficiency towards electrochemical capacitance. However, modifying the surface by creating porosity, inducing vacancy and modifying with some other materials improve their activity. In this section the GaN and GaN-based electrode materials towards supercapacitor activity will be discussed.

**Fig. 4:**
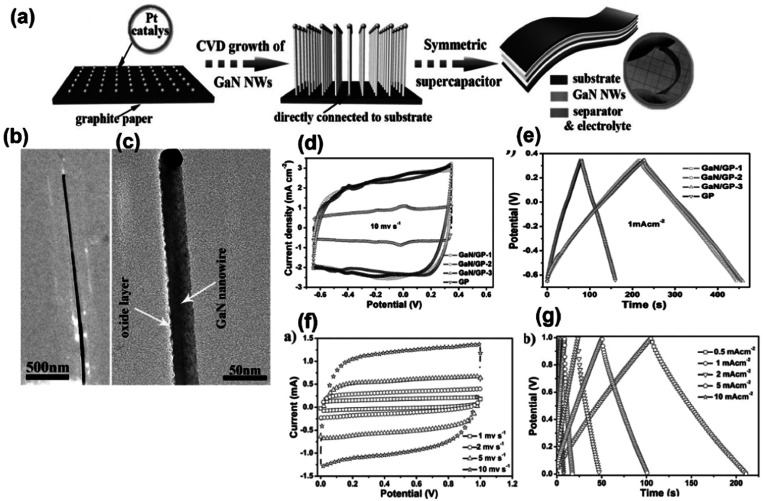
(a) Synthesis process of GaN/GP and its assembly into symmetric supercapacitors. (b and c) TEM image of GaN NW. (d) CV graph at a sweep rate of 10 mV s^−1^. (e) GCD plot at a current density of 1 mA cm^−2^ in the three-electrode set up. (f) CV curves and (g) GCD profiles of symmetric all-solid-state SCs fabricated by coupling hybrid GaN/GP-2 electrodes (this figure has been reproduced from ref. 26 with permission from Wiley, copyright 2002).

**Fig. 5:**
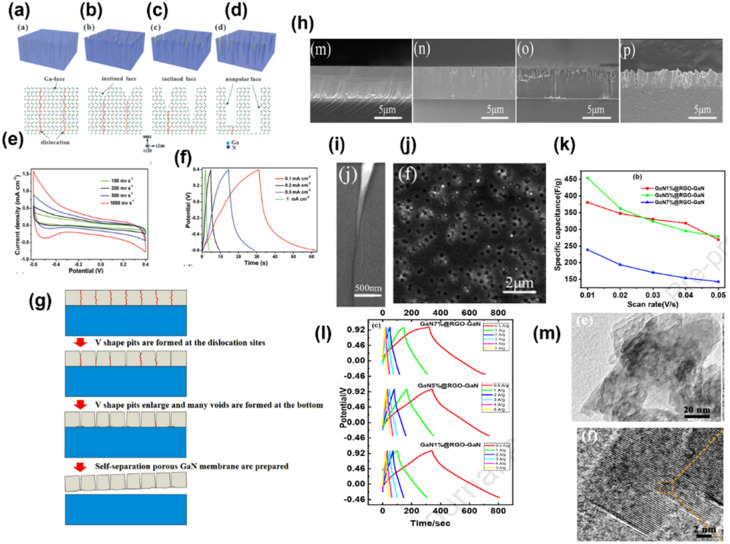
(a–d) Illustration of the formation method of the GaN porous template. Electrochemical performance of the porous GaN electrode. (e) CV and (f) GCD plots (these (a–f) have been reproduced from ref. 27 with permission from RSC, copyright 2016). (g) Separation model to fabricate GaNPM. (h) SEM images of the GaNPM formation. (i) TEM image of the V-shaped pit. (j) CL image of porous GaN (1200 °C for 30 min) ((g–i) have been reproduced from ref. 28 with permission from Nature, copyright 2017). RGO-GaN nanocomposites (GaN1%@RGO-GaN, GaN5%@RGO-GaN and GaN7%@RGO-GaN): (k) plot between the scan rate *vs.* specific capacitance and (l) charge–discharge curves ((k and l) have been reproduced from ref. 29 with permission from Elsevier, copyright 2020). (m) TEM and HRTEM images of GaN-2.5 h (this figure has been reproduced from ref. 30 with permission from RSC, copyright 2022).

Wang *et al.* synthesized a single-crystal GaN mesoporous membrane (GaNMM) by adopting an electrochemical etching method to study its application towards supercapacitors for the first time. The mesoporous structure of the GaNMM was confirmed by SAXRD from the appearance of a sharp peak at a 2*θ* value of 0.42° with a *d*_(100)_ spacing value of 21 nm. The result is coincident with the BET analysis and pore size obtained from SEM images. The mesoporous nature enhanced the electrochemical properties by rapid electrolyte transport to its pore, and the defect site provided more active sites for chemical reactions. This results in good specific capacitance with cycling performance.^25^ In another work, with the intention of enhancing the capacitance of GaN, they (2017) fabricated GaN nanowires (GaNNWs)/graphite paper (GP) as a high-performance flexible supercapacitor electrode. The enhancement in the electrochemical properties of the composite is due to their outstanding electrical conductivity (6.36 × 10^2^ S m^−1^ and GP: 7.5 × 10^4^ S m^−1^), which was verified by the Hall effect analysis. Fig. 4a illustrates the preparation process of GaN/GP and its assembly into a flexible symmetric supercapacitor. Fig. 4b and c present the TEM images of GaN/GP-2 (GaN/GP-*x* composite *x* = 1, 2, and 3 w.r.t. their growth time of 20, 30, and 40 min, respectively), which consists of a cylindrical structure with a uniform diameter. The vapour–liquid–solid mechanism is followed for the growth of GaN NW, which is indicated by the presence of Pt nanoparticles at the end of the GaN NWs. In Fig. 4b, the single-crystalline nature of the GaN NW without any other subordinate phase is observed. Fig. 4d and e present the CV (at 10 mV s^−1^) and GCD (1 mA cm^−2^) curves of both GP and GaN/GP respectively, which clearly indicated pseudocapacitance along with double-layer influence. Fig. 4f and g show the electrochemical performance of symmetric all-solid-state SCs fabricated by coupling hybrid GaN/GP-2 electrodes in an H_2_SO_4_/PVA gel. The areal capacitance was calculated from CV to be 55.8 m^2^ cm^−3^ at a sweep rate of 55.8 mF cm^−3^ at a scan rate of 1 m cm^−1^ with 68.6% of capacitance retention at a scan rate of 200 mV s^−1^. The areal and volumetric capacitances were found to be 53.2 mF cm^−2^ and 21 F cm^−3^ at a current density of 6.5 mA cm^−2^. They found that the device is flexible, as there is no noticeable variation in the cyclic performance under different bending conditions.^26^ In another work, Zhang group adopted a novel high-temperature annealing process (annealing at 1150 °C for different times 30, 45, 60, 75, 90 and 105 minutes) to produce wafer-scale (2 inch) porous GaN. The crystal quality of GaN was enhanced *via* the high-temperature annealing process, as indicated by Raman, PL and HRXRD characterization. Based on Cebrera's thermodynamic theory, a model has been proposed, which explained the formation mechanism of porous GaN by a high-temperature annealing process, as shown in Fig. 5a. According to the mechanism, decomposition first occurs at the dislocation site of the Ga face to form V-shaped pits; with annealing time, V shaped pit decomposed along the vertical direction, as shown in Fig. 5b–d. With the further increase in time, porous GaN was detached steadily and slowly resulted in the exposure of non-polar face. Therefore, rectangular pits were formed owing to the stability of the Ga face and non-polar face. The electrochemical analysis of the porous GaN electrode is shown in Fig. 5e and f. Based on this, it can be said that the porous GaN-based electrode shows good capacitive behavior (3.12 mF cm^−2^ at 0.1 mA cm^−2^) with good electrochemical reversibility, and hence, it can be considered a potential candidate for supercapacitors.^27^ In connection to the above-mentioned work, Zhang group fabricated a single-crystalline porous GaN membrane (GaNPM) by adopting a one-step high-temperature annealing method (1200 °C for 30–80 minutes).^28^ They proposed a model to show the separation process of GaNPM based on the thermodynamic theories, as shown in Fig. 5g. Fig. 5h–j show the SEM, HRTEM, and CL spectra, which are in support of the proposed model. The crystallinity of the self-separated GaNPM was confirmed by HRTEM. Fig. 5i displays the cross-sectional TEM images of the V-shaped pit formed towards dislocation, the same as the predicted model. The secondary electron and CL image of GaNPM is shown in Fig. 5j. The GaNPM-based asymmetric supercapacitor shows comparable areal power density to that of EDLCs such as graphenes, Ti-CNTs, and GaN membranes, as well as pseudocapacitors such as ZnO/MoO_2_, polyaniline, GN@MnO_2_, and N-doped SiC.^28^ Nongthombam *et al.* prepared rGO-GaN nanocomposites by a facile chemical reduction process, in which they varied the concentration of GaN (from 1 wt% to 20 wt%) w.r.t. rGO.^29^ The formation of composites was confirmed by XRD analysis and SEM images. From XPS analysis, the calculated atomic % ratio of Ga and N for GaN 1%@ rGO-GaN and GaN 7%@rGO-GaN was found to be in the range of 2.32–3.98% and 4.91–7.33% respectively. The incorporation of GaN increases the surface pores, leading to an enhancement in the specific capacitance of the nanocomposites. The maximum specific capacitance of the rGO-GaN nanocomposite was found to be 454 F g^−1^ at 10 mV s^−1^ scan rate for 5%@rGO-GaN using cyclic voltammetry results; and a similar trend was also observed in a galvanostatic charge–discharge method, as shown in Fig. 5k and l.^29^ Lv *et al.* prepared a vacancy-modified few-layered GaN crystal *via* a one-step ball milling process, and it was first applied in a high-temperature energy storage field. The constructed IL (ionic liquid)-based supercapacitor device exhibited excellent electrochemical performance even at an ultra-high temperature of 150 °C, and ILs successfully suppressed the probability of side reactions and volume expansion of the devices; therefore, it can be useful under extreme working conditions. From the TEM images shown in Fig. 5m, it can be found that the GaN crystal consists of transparent similar layers that restrict the self-aggregation of materials through the course of energy storage. Moreover, this ultrathin structure offers a large surface area with more active sites (N-vacancies), resulting in capacitance enhancement. Further, they claimed that this work has opened up a new avenue for the application of GaN crystal materials.^30^

### 3.2 GaON-based supercapacitors

**Fig. 6:**
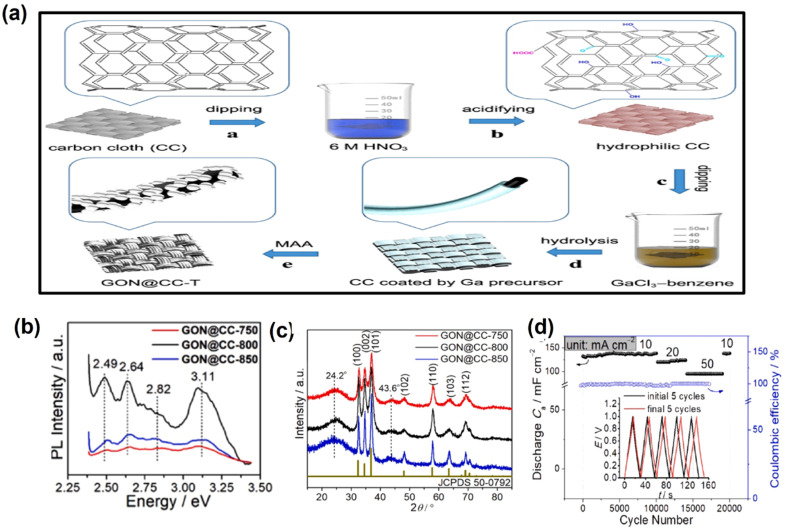
(a) Synthesis procedure of GaON@CC-T. (b) Room-temperature PL spectra and (c) XRD spectra of GaON@CC-T (*T* = 750 °C, 800 °C, and 850 °C). (d) Capacitance retention of the symmetric supercapacitor fabricated with GaON@CC-800 (these figures have been reproduced from ref. 31 with permission from Elsevier, copyright 2020) (the caption “GON” mentioned in the figure is the same as “GaON”).

Metal oxynitride (MON) refers to the partial substitution of O atoms by N atoms in metal oxides, which results in the formation of a new material, *i.e.* metal oxynitride. GaON as an electrode material is considered to be the emerging candidate for the energy conversion and storage process. However, GaON with the phase resembling wurtzite gallium nitride (GaN) was found to be a propitious electrode material for supercapacitors. Wang *et al.* prepared binder-free GaON@CC (GaON nanoparticles grown on carbon cloth) using GaCl_3_ as the gallium source *via* an MAA approach (moisture-assisted ammonolysis), as shown in Fig. 6a. In the presence of moisture, kinetics and thermodynamics of the system are influenced during the nitridation process, thereby increasing the physicochemical properties of the semiconductor. Further, it was found that by controlling the nitridation temperature, the particle size, lattice parameters, N/O ratio, band structure and defect site of the GaON@CC can be tuned. Among all the GaON@CC electrodes, the material subjected to nitridation at 800 °C, *i.e.* GaON@CC-800, exhibits enhanced electrochemical performance in comparison to others, which may be due to the presence of relatively more defect sites and the lengthened Ga–N bond, which is confirmed from the PL spectra and XRD analysis, as shown in Fig. 6b and c. The symmetric supercapacitor fabricated with GaON@CC-800 shows 100% capacitance retention (initial capacitance 132 mF cm^−2^ at 10 mA cm^−2^) after 20 000 cycles, as shown in Fig. 6d. This work demonstrated with theoretical and experimental evidence that by controlling the N/O ratio, the electrochemical performances of the electrode material can be manipulated.^31^ In another work, Wang group prepared GaON nanoparticles by an ammonolysis technique and used them as electrode materials for supercapacitors. While working on GaON nanoparticles, they found that pristine GaON exhibits a relatively low electrochemical performance towards energy storage. In order to enhance the electrochemical activity, they created gallium vacancies (*V*_Ga_) and anion defects into GaON nanoparticles *via* a controllable electrochemical etching process. However, the textural property, defect concentration and band structures can be manipulated by controlling the etching time. Based on experimental as well as computational outcomes, they described the effect of both cation and anion defects on the electrochemical performances. The enhanced performance of the etched GaON nanoparticle is due to the (i) removal of surface oxide layer, (ii) improvement in specific surface area (*S*_BET_) and pore volume (*V*_pore_) and (iii) engineered cation and anion vacancies. Furthermore, they found out that the etching process did not compromise with the material's cycling stability and rate capability.^32^

### 3.3 LDH-based supercapacitors

**Fig. 7:**
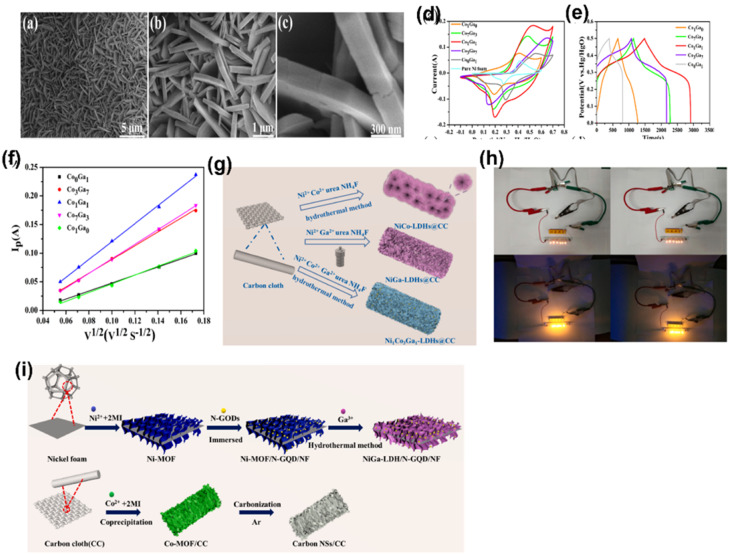
(a–c) SEM images of the CoGa-LDH with different feeding ratios of Ga/Co (*i.e.* 7 : 3, 1 : 1 and 3 : 7), respectively. (d) CV curves and (e) GCD curves of different feeding ratios of CoGa-LDH. (f) Plot between *I*_p_ and *ν*^1/2^ ((a–f) have been reproduced from ref. 33 with permission from Elsevier, copyright 2022). (g) Fabrication process of NiCo-LDH@CC, NiGa-LDH@CC and NiCoGaLDH@CC. (h) Two devices connected in series light five LEDs in parallel ((g and h) have been reproduced from ref. 35 with permission from Elsevier, copyright 2021). (i) Preparation method of NiGa-LDH/N-GQD/NF and carbon nano sheets (this figure has been reproduced from ref. 36 with permission from Elsevier, copyright 2022).

LDHs are deserved 2D lamellar and dendritic structured electrode materials for supercapacitors due to its high availability, reversible redox reaction, good theoretical conductance, variation in morphologies, and *in situ* incorporation of different cations. However, the inherent properties are low conductivity and poor stability during redox activity, which could be enhanced by the formation of composites with carbonaceous materials. X. Chen *et al.* for the first time developed a hierarchical flower-sheet CoGa LDH from cobalt nitrate hexahydrate, gallium nitrate, urea and ethanol used as precursors, which was *in situ* grown on a Ni foam *via* a hydrothermal route. A different ratio of Co to Ga was introduced to study the morphology and capacitive nature.^33^ The authors showed different morphologies for different Co/Ga ratios by electron microscopy. They finally concluded about different metal substitutions play an important role of morphology and structure variation since Co^2+^ was completely affixed in Ga^3+^ avoiding agglomeration due to the creation of excess positive charges, as shown in Fig. 7a–c. The electrochemical properties of the CoGa LDH were evaluated using a three-electrode system. Different feeding ratios of Co/Ga (*i.e.* 3 : 7, 1 : 1 and 7 : 3) of the CoGaLDH were used to optimize the performance. Fig. 7d and e represent the CV and GCD curves of the CoGaLDH with different feeding ratios at a scan rate of 30 mv s^−1^. The integral area of CV for the Co_1_Ga_1_LDH was found to be more than that of the LDH with other feeding ratios, which indicates better supercapacitive activity. Furthermore, they found the distinctive redox peaks which demonstrate the reversible and battery capacity properties of the LDH. From the GCD curves, the specific capacity of Co_1_Ga_1_LDH was calculated by the author and found to be 1431.4 C g^−1^ at a current density of 20 A g^−1^. From Fig. 7f, the linear relationship among peak current (*I*_p_) and the square root of scan rates (*ν*^1/2^) was found, which indicates that the electrochemical reaction occurs *via* diffusion-controlled processes. In order to inspect the practicability of the device, a hybrid supercapacitor was assembled by taking Co_1_Ga_1_LDH as the positive electrode and rGO as the negative electrode respectively. The device showed a large operation potential window of 1.65 V with a high energy density of 68.07 W h kg^−1^ at a power density of 825 W kg^−1^. By considering the facile synthesis method and superior electrochemical performance, it is concluded that the CoGa-LDH may be the promising electrode material in the field of energy storage.^33^ Zhang *et al.* prepared an oxygen-defect-rich 3D porous CoGaLDH (Co_0.5_Ga_0.5_-LDH) for high-performance supercapacitor applications by a simple one-step method.^34^ They quickly poured an aqueous solution containing Co^2+^ and Ga^3+^ into an aqueous solution of hexamethylenetetramine followed by a mild and fast hydrothermal reaction. Further, the group claimed that by adopting a mild and rapid approach, a large number of pores were introduced into the ultrathin LDH nanosheets, which result in the creation of a large number of oxygen vacancies in the CoGaLDH. The oxygen vacancies can be arbitrarily controlled, which was confirmed from XPS and ESR measurements. The group concluded that the synergistic effect of introducing Ga ions into the LDH and oxygen vacancies improves the adsorption of the LDH nanosheets on OH-, which results in the outstanding performance of the Co_0.5_Ga_0.5_-LDH towards supercapacitor applications. In order to test the practical application of Co_0.5_Ga_0.5_-LDH, aqueous ASC (asymmetric supercapacitor) using Co_0.5_Ga_0.5_-LDH as the positive electrode and activated carbon as the negative electrode has been fabricated. The device delivered an ultrahigh energy density of 33.38 W h kg^−1^ and a power density of 10 088 W kg^−1^. Finally, it was claimed that this work would provide deep insights into the outcome of oxygen vacancies that lower the *E*_ads_ OH^.^ of LDHs and encourage the wide application of 3D porous ultrathin LDH nanosheets towards energy storage.^34^ Li *et al.* fabricated a Ga(ii)-doped NiCo LDH (*i.e.* NiCoGa-LDH@CC) on carbon cloth by a simple one-step hydrothermal method for the first time.^35^ Similarly, NiCo-LDH@CC and NiGa-LDH@CC have been prepared in order to compare the activity with NiCoGa-LDH@CC. Fig. 7g presents the synthesis strategy of NiCo-LDH@CC, NiGa-LDH@CC and NiCoGa-LDH@CC. The group systematically investigated the effect of Ga^3+^ doping on the structure, morphology and electrochemical performance of the ternary LDH, *i.e.* NiCoGa-LDH@CC. Further, it has been found that with the increase in the doping concentration of Ga^3+^, the dynamic of ion diffusion accelerates due to the opening in the interlaminar space. With the increase in Ga^3+^ concentration, more redox active sites are formed, which leads to the degradation of crystalline nature and morphology, ultimately inducing negative impacts on the electrochemical performance. With Ga^3+^ = 1; the synthesized LDH, *i.e.* Ni_1_Co_3_Ga_1_-LDH@CC, shows the highest specific capacitance of 2012.5 F g^−1^ at 1 A g^−1^ with 57.7% capacitance retention observed at 5 A g^−1^. Further, when the ASC (asymmetric supercapacitor) device was assembled using Ni_1_Co_3_Ga_1_-LDH as the anode and MPC (mesoporous carbon) as the cathode, the system exhibited 84.22 W h kg^−1^ of energy density at 800.1 W kg^−1^ of power density. To further test the practical application, two devices were connected in series to light five LEDs in parallel, as shown in Fig. 7h. This work offers a new way to enhance the electrochemical stability and performance of ternary LDHs.^35^ In another work, Li group prepared nitrogen-doped graphene quantum dots for high-performance asymmetric supercapacitors. It is well known that due to the multiple oxidation states, the LDH is a promising material for supercapacitors. However, it is susceptible to breakdown during redox reactions and exhibits low conductivity, which hampers its electrochemical performance. Li group prepared interlaced nanosheet hybrid structures by assembling N-GQDs (nitrogen-doped graphene quantum dots) with the NiGa-LDH derived from a 2D Ni-MOF on a Ni foam denoted as NiGa-LDH/N-GQD/NF. The specific capacitance of NiGa-LDH/N-GQD/NF was found to be 2160 F g^−1^ at 1 A g^−1^ with 87.5% of capacitance retention after 5000 cycles. In addition, ASC devices with NiGa-LDH/N-GQD/NF as the cathode and carbon nanosheets as the anode was fabricated. The preparation method of NiGa-LDH/N-GQD/NF and carbon nanosheets is shown in Fig. 7i. The device shows an energy density of 8.8 W h kg^−1^ at a power density of 1432.7 W kg^−1^. In order to demonstrate the practical application, several LED lights were lit. The group claimed that this work would pave a new pathway for the fabrication of LDHs based on MOF-encapsulated N-doped quantum dot hybrid electrodes for electrochemical energy storage applications.^36^

### 3.4 Transition metal oxide-based spinel supercapacitors

**Fig. 8:**
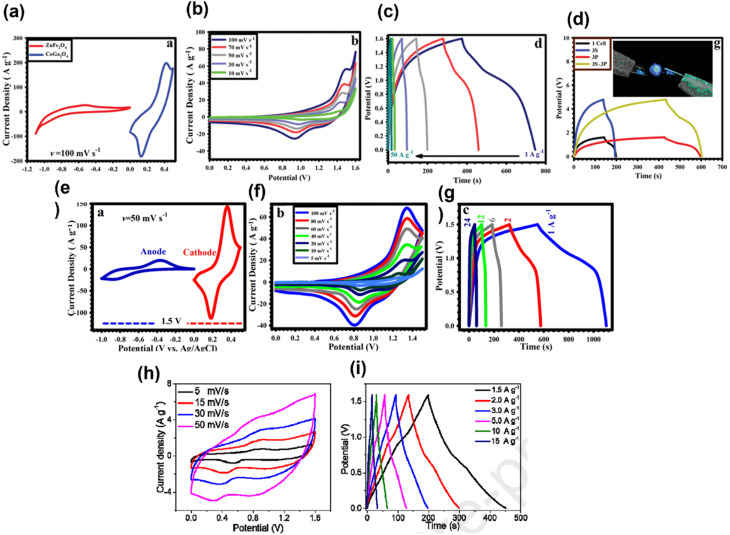
(a) CV plots of the CoGa_2_O_4_/Ni foam and ZnFe_2_O_4_/Ni foam electrode at 100 mV s^−1^ in a 2 M KOH solution. (b) CV curves of the device. (c) CD curves of the device. (d) CD curves of the devices coupled in series and in parallel ((a–d) have been reproduced from ref. 37 with permission from RSC, copyright 2019). (e) CV plots of the positive and negative electrodes at 50 mV s^−1^ (f) CV plots of HTS-CGOHS//GW@CFSHS at different scan rates. (g) CD plots of HTSCGOHS//GW@CFSHS at various current densities ((e–g) have been reproduced from ref. 38 with permission from ACS, copyright 2019). (h) CV curve of the device in the potential range of 0–1.6 V at various scan rates. (i) GCD curves at various current densities ((h and i) have been reproduced from ref. 39 with permission from Elsevier, copyright 2020).

Transition metal oxides *via* rapid faradic redox reactions lead to high energy density compared to EDLCs. Due to high theoretical specific capacitance and high environmental compatibility, Co-based spinel materials with the general formula CoM_2_O_4_ (M = Mn and Fe) are extensively studied in the field of supercapacitor. CoM_2_O_4_ suffers from low cycling stability, rate capability, and electronic conductivity but after introducing Ga into spinel materials, enhancement in the storage properties of the cathode material is seen. CoGa_2_O_4_ is much focused in the area of electrochemical energy storage owing to its properties such as rich redox reactions, high stability, chemical compatibility with Ni foams and high specific capacitance. Zardkhoshousi *et al.* for the first time synthesized CoGa_2_O_4_ on a Ni foam by adopting a facile electrodeposition method and used it as the cathode (positive electrode) for asymmetric supercapacitors (specific capacitance of CoGa_2_O_4_: 1379.16 F g^−1^ at 50 A g^−1^).^37^ For anode materials, ZnFe_2_O_4_ fabricated on a Ni foam by a electrodeposition method was selected (specific capacitance: 300.5 F g^−1^ at 50 A g^−1^). When an asymmetric supercapacitor was assembled using CoGa_2_O_4_ as the cathode (positive electrode) and ZnFe_2_O_4_ as the anode (negative electrode), a noteworthy electrochemical performance is seen (specific capacitance; 232.2 F g^−1^ at 1 A g; cyclic retention: 64%, 6000 cycles, 50 A g^−1^). Fig. 8a presents the CV plots of the CoGa_2_O_4_/Ni foam and ZnFe_2_O_4_/Ni foam electrode at 100 mV s^−1^ in a 2 M KOH solution. The voltage window of CoGa_2_O_4_ is 0 to 0.5 V and for ZnFe_2_O_4_ it is −1.1 to 0. The total device voltage is expressed as the sum of the voltage of the ZnFe_2_O_4_/Ni foam and CoGa_2_O_4_/Ni foam, *i.e.*, 1.6 V. Fig. 8b and c present the CV curves of the devices at various sweep rates and CV curves at various current densities. Fig. 8d presents the GCD curves of the devices connected in series and in parallel. As this can light the blue LED indicator after connecting the device together, it can be used in future electronic systems. Further, it has been stated that this type of work could produce new materials for next-generation storage applications.^37^ In another work, Zardkhoshoui *et al.* developed an asymmetric supercapacitor by taking a highly porous triple-shelled CoGa_2_O_4_ hollow sphere (HTS-CGOHS) with a triple narrow shell as the positive electrode and a pseudocapacitance graphene-wrapped CuFeS_2_ hollow sphere (GW@CFSHS) as the negative electrode.^38^Fig. 8e presents the CV plots of positive and negative electrodes at 50 mV s^−1^; after charge balancing, the voltage window of electrodes was found to be 0 to 0.50 V and −1 to 0 V respectively. Fig. 8f presents the CV plots of HTS-CGOHS//GW@CFSHS at different scan rates with the potential windows of 0 to 1.5 V. As the potential window of HTS-CGOHS goes up to 1.5 V with the KOH electrolyte. The electronic as well as ionic transmission in the electrodes results in sharp retention in all scan rates, leading to excellent rate performance. The approximately symmetric charge/discharge nature of the CD curve and the excellent coulombic efficiency of the asymmetric supercapacitor are revealed by the CD curve shown in Fig. 8g. This work deals with the promising strategy for the fabrication of cutting-edge electrode materials for supercapacitors and confirmed the potential application of devices in next-generation energy storage devices.^38^ Javed *et al.* prepared a high-energy electrode of asymmetric supercapacitors by growing a Co–Ga hexagonal nano plate on a flexible carbon cloth (CC) substrate.^39^ The optimized electrode exhibits good electrochemical storage performance (1525 F g^−1^ at 5 A g^−1^), investigated using electroanalytical methods and ex situ XPS. In order to know the real potential of the electrode, an all-solid-state asymmetric supercapacitor was fabricated by taking CoGa_2_O_4_@CC as the positive electrode and activated carbon supported on CC, *i.e.*, AC@CC, as the negative electrode. The asymmetric cell is denoted as CoGa_2_O_4_@CC//AC@CC. A quasi-solid-state gel was used to assemble the device. Fig. 8h presents the CV curve of the device in the potential range of 0–1.6 V at various scan rates, and Fig. 8i presents the GCD curves at various current densities. The specific capacitance of the device was found to be 239 F g^−1^ at 1.5 A g^−1^. The device shows admirable results, which may be ascribed to the typical ultra-fast ionic diffusion and higher surface area, leading to an increase in the number of active sites and shortening the diffusion path among ions and electrodes.^39^

### 3.5 Other systems

**Fig. 9:**
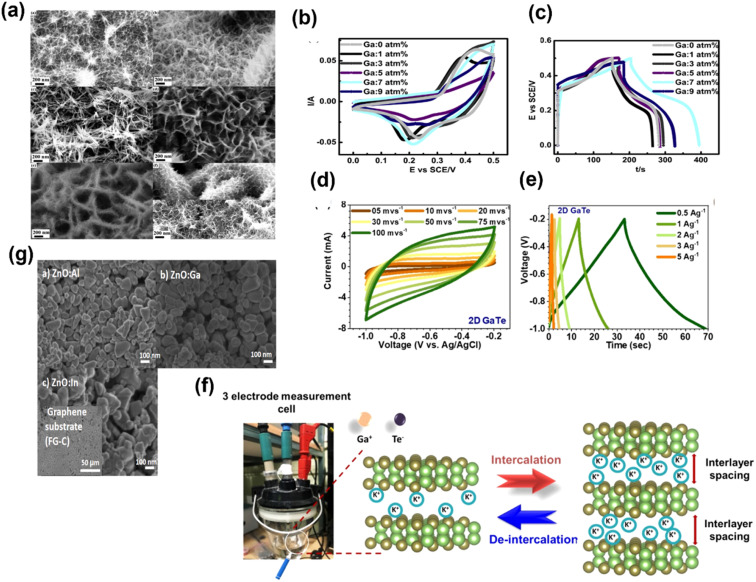
(a) SEM images for change in the nano wired structure of NiO after the incorporation of Ga: (A) 0 atm%, (B) 1 atm%, (C) 3 atm%, (D) 5 atm%, (E) 7 atm%, and (F) 9 atm%. (b) CV and (c) GCD curves of Ga-incorporated nickel oxide ((a andb) have been reproduced from ref. 40 with permission from Elsevier, copyright 2016). (d) CV and (e) GCD curves of 2D GaTe at various scan rates and current densities. (f) Intercalation and deintercalation processes of 2D GaTe ((d–f) have been reproduced from ref. 41 with permission from ACS, copyright 2021). (g) SEM images of (a) ZnO:Al, (b) ZnO:Ga and (c) ZnO:In (this figure has been reproduced from ref. 42 with permission from Elsevier, copyright 2022).

**Fig. 10:**
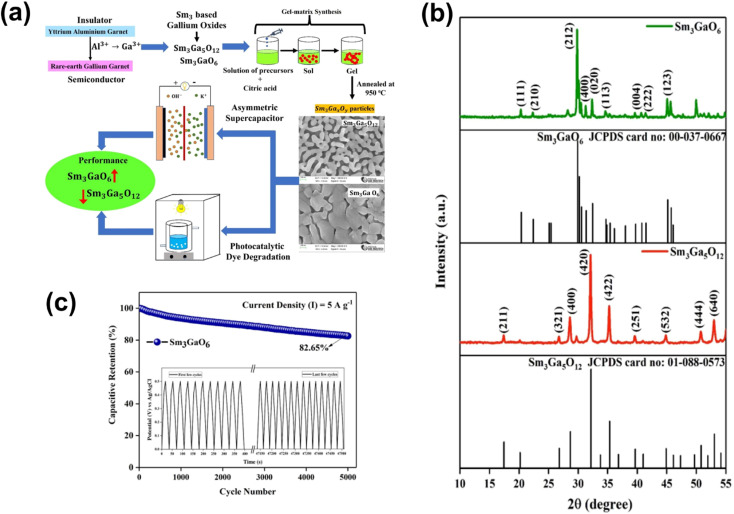
(a) Synthesis process of Sm_3_Ga_*x*_O_*y*_. (b) XRD spectra of Sm_3_GaO_6_ and Sm_3_Ga_5_O_12_. (c) Cycling stability curve of Sm_3_GaO_6_ ((a–c) have been reproduced from ref. 43 with permission from RSC, copyright 2023).

The logical approach for enhancing the conductivity and supercapacitor performance is to dope p-type materials with p-type acceptors. However, Zhang *et al.* incorporated p-type NiO with an n-type element. In that study, Group III redox inactive element Ga has been selected to incorporate into NiO with different atomic ratios (0 atm%, 1 atm%, 3 atm%, 5 atm%, 7 atm% and 9 atm%), and the effects of incorporation on the crystal structure and supercapacitor performance have been studied.^40^ The irregular alteration of morphology and performance after the incorporation of Ga into NiO was found. Fig. 9a shows the SEM images for change in the nano wired structure of NiO after the incorporation of Ga. Fig. 9b presents the CV curves of Ga 0 (pure NiO), Ga 1(Ga: 1 atm%), Ga 3 (Ga: 3 atm%), Ga 5 (Ga: 1 atm%), Ga 7 (Ga: 7 atm%) and Ga 9 (Ga: 9 atm%) in 1 KOH at 5 mV s^−1^. As Ga has only one oxidation state *i.e.*, +3, it will not contribute towards redox reactions. Furthermore, it will lower the p-type conductivity, resulting in low performance. However, interestingly, the group found that the highest current was obtained for Ga 7 and the lowest for Ga 9 compared to Ga 0, as displayed in Fig. 9b. The GCD curves of the materials at a current density of 7.5 mA cm^−2^ are presented in Fig. 9c. Similarly, Ga 7 shows the highest discharging time.^40^ Siddique group proposed an easily scalable fabrication method by ultrasonication-assisted liquid exfoliation in a solvent medium of 2-propanol to obtain atomically thin GaTe (gallium telluride) and claimed that this work will open up the possibility of application of this well-known material.^41^ Further, the construction of atomically thin sheets (thickness ∼2 nm) was confirmed by AFM, TEM and Raman spectroscopy. From the X-ray diffraction study (XRD), the lattice rearrangement as a function of reducing size was found. Furthermore, the transformation of the monoclinic structure to the hexagonal crystal structure after exfoliation is seen. The hexagonal crystal structure was confirmed from the HRTEM images, and it also confirmed the presence of surface defects. Fig. 9d and e present the CV and GCD curves of 2D GaTe at various scan rates and current densities, respectively. The specific capacitance of 2D GaTe shows high stability (96% cyclic retention, 10 000 cycles at 1 A g^−1^), which may be due to the opening of pores at the surface of the electrode during intercalation and deintercalation processes, as schematically represented in Fig. 9f. With this work, Siddique group supported the possible use of GaTe in optoelectronic and energy storage applications.^41^ Badilo *et al.* enhanced the electrochemical performance of graphene supercapacitors by coating the electrode with one of the slurry-paste of ZnO:Al, ZnO:Ga and ZnO:In.^42^ The group claimed that the use of doped Zn in supercapacitors has not been reported previously.^42^ They found that after doping ZnO, the oxygen vacancy created in its crystalline lattice becomes beneficial for its electrochemical performance. The SEM images of ZnO:Al, ZnO:In and ZnO:Ga are shown in Fig. 9g, from which the plate-like morphology with irregular edges was found with hexagonal wurtzite phase. The enhancement in the capacitance and energy density up to 193% w.r.t. the reference device without ZnO was found. The maximum value of capacitance and energy density was found for the device made with ZnO:Al, *i.e.*, 284.6 F g^−1^ and 56.9 W h kg^−1^. By analyzing the Raman and photoluminescence, the presence of Zn interstitial oxygen vacancies and defects that act as the redox center for charge storage in the device has been demonstrated. This work opened up a new avenue for the researchers by adding doped ZnO into the supercapacitor device to enhance the device performance.^42^ Sm_3_Ga_*x*_O_*y*_ (samarium gallium oxide) is a complex material system; depending upon the composition and synthesis conditions, this can have multiple phases.^43^ A cutting-edge development has been attempted by Shanmugam *et al.* for the synthesis of two stable phases of samarium gallium oxide for photosynthesis and energy storage electrodes. A gel matrix method has been adopted for the synthesis of two stable phases, as represented in Fig. 10a. Fig. 10b presents the XRD peaks of Sm_3_GaO_6_ and Sm_3_Ga_5_O_12_ with the corresponding cubic and orthorhombic phases with no other impurities. Fig. 10a presents the FESEM images of both the phases having self-assembled nano-structures. The specific capacitance of Sm_3_GaO_6_ (103.89 mA h g^−1^ at 1 A g^−1^) was found to be higher than that of Sm_3_Ga_5_O_12_ (91.95 mA h g^−1^ at 1 A g^−1^) with significant cycling stability and a capacitive retention of 82.65% over 5000 cycles at 5 A g^−1^, as shown in Fig. 10c. The photocatalytic studies for methylene blue degradation were also performed for two phases and the degradation efficiency was found to be 92% and 97% for Sm_3_Ga_5_O_12_ and Sm_3_GaO_6_ respectively, within 2 h of light irradiation. Finally, it was concluded that the both phases of samarium gallium oxide have the potential for energy storage and environmental remediation applications.^43^

## 4. Challenges and future perspectives

Gallium is a group III element that has only one oxidation state *i.e.*, +3; therefore, it generally does not take part in redox reactions and majorly contributes towards pseudocapacitive behaviours. Despite redox inactiveness, gallium-based materials have shown excellent electrochemical activity towards supercapacitor applications, and the development of gallium-based electrodes is in its infant stage. Thus, numerous efforts should be made for the development of gallium-based electrodes by improving their intrinsic charge storage mechanism, which can augment the performance of advanced energy storage devices. Thus, gallium-based materials in supercapacitor applications have the potential to replace traditional energy storage technologies such as Li-ion batteries owing to several advantages such as high power density, long cycle life, stability over a wide range of temperatures, and fast charge and discharge rate. Furthermore, they also have the potential for high energy density upon further modification. The followings are few challenges and future perspectives of gallium-based materials towards supercapacitor applications:

(i) Cost-effectiveness and scalability: scalability and cost-effectiveness are some of the major challenges faced by gallium-based materials, which hinder their practical applications. Large-scale production by adopting environmentally friendly methods should be subjected to further research for the development of gallium-based materials.

(ii) Integration with advanced storage systems: to cope with the high energy storage requirements, gallium-based supercapacitors can be integrated with other advanced storage systems such as Li-ion batteries, hybrid systems or fuel cells to increase the overall storage performance. This integration may increase the energy density and power density of the energy system. The optimization of the design of integrated energy storage systems and the exploration of their synergistic effects should be the topic of future research.

(iii) Sustainability and environmental aspects: as the field of energy storage is advancing, it is necessary to monitor the sustainability and environmental aspects of the electrode materials. The synthesis method, electrode materials, electrolytes, packaging of the device and recycling process should be sustainable and environmentally friendly. The focus of the future research should be on the development of green synthesis methods for gallium-based electrodes with suitable green electrolytes with excellent recycling strategies.

(iv) Enhanced energy density: researchers are progressively exploring new gallium-based electrodes to enhance the energy storage performance, *i.e.*, energy density for supercapacitor activity. The energy storage capacity of gallium-based supercapacitors can be significantly enhanced by nanostructuring the electrodes and optimizing the electrolyte composition.

(v) Efficiency: the efficiency of gallium-based electrodes can be improved by advances in materials science, reducing the internal energy, electrode engineering and device architecture.

(vi) Market adaptation and commercialization: the commercialization and market adaptation of any system mainly depends upon several factors including performance and efficiency, cost scalability, safety, regulatory environment and market demand. The gallium-based supercapacitors are still in their initial stage of research and they have the potential for market adaptation and commercialization in the future.

**Scheme 2:**
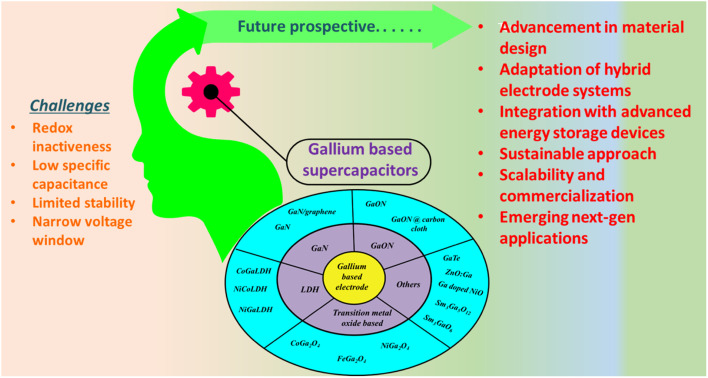
Various gallium-based systems for supercapacitors.

Overall, gallium-based supercapacitors possess a substantial potential for advancing supercapacitor technologies and to address the limitations of traditional electrode materials. Further, extensive research in this field could lead to the development of high-performance supercapacitors addressing the above-mentioned challenges (Scheme 2).

## Conflicts of interest

There is no conflict of interest to declare.

## Abbreviation

ESSs: Electrochemical energy storage systems
EDLC: Electric double-layer capacitance
TMO: Transition metal oxide
TMS: Transition metal sulfide
SCs: Supercapacitors
PC: Pseudocapacitance
CV: Cyclic voltammetry
GCD: Galvanostatic charge discharge
SAXRD: small-angle X-ray diffraction
SEM: Scanning electron microscopy
FESEM: Field emission scanning electron microscopy
TEM: Transmission electron microscopy
HRTEM: High-resolution transmission electron microscopy
XRD: X-ray diffraction
HRXRD: High-resolution X-ray diffraction
CL: Cathodoluminescence
IL: Ionic liquid
PL: Photoluminescence
LDH: Layered double hydroxide
XPS: X-ray photoelectron spectroscopy
ESR: Electron spin resolution
ASC: Asymmetric supercapacitor
AFM: Atomic force microscopy

## Supplementary Material
